# The composite autonomic symptom scale 31 is a useful screening tool for patients with Parkinsonism

**DOI:** 10.1371/journal.pone.0180744

**Published:** 2017-07-06

**Authors:** Younsoo Kim, Jin Myoung Seok, Jongkyu Park, Kun-Hyun Kim, Ju-Hong Min, Jin Whan Cho, Suyeon Park, Hyun-jin Kim, Byoung Joon Kim, Jinyoung Youn

**Affiliations:** 1 Department of Neurology, Samsung Changwon Hospital, Sungkyunkwan University School of Medicine, Changwon, Korea; 2 Department of Neurology, Soonchunhyang University Cheonan Hospital, Soonchunhyang University College of Medicine, Cheonan, Korea; 3 Department of Neurology, Soonchunhyang University, Gumi, Korea; 4 Department of Neurology, Samsung Medical Center, Sungkyunkwan University School of Medicine, Seoul, Korea; 5 Neuroscience Center, Samsung Medical Center, Seoul, Korea; 6 Department of Biostatistics, Soonchunhyang University Seoul Hospital, Soonchunhyang University College of Medicine, Seoul, Korea; University of Pennsylvania Perelman School of Medicine, UNITED STATES

## Abstract

Differentiation of multiple system atrophy with predominant parkinsonism (MSA-P) and Parkinson's disease (PD) is important, but an effective tool for differentiation has not been identified. We investigated the efficacy of the composite autonomic symptom scale 31 (COMPASS 31) questionnaire as a tool for evaluating autonomic function in parkinsonism patients. In this study, we enrolled drug-naïve patients with MSA-P and PD, and administered the COMPASS-31 and an objective autonomic dysfunction test (AFT). Demographic and clinical data, including parkinsonism and autonomic dysfunction, were compared between the two groups. Additionally, we determined the optimal COMPASS 31 cut-off score to differentiate MSA-P from PD for use as a screening tool. In this study, 27 MSA-P patients and 41 PD patients were recruited. The total COMPASS 31 score was well correlated with the objective AFT results. When we compared the COMPASS 31 score between the two groups, MSA-P patients showed higher total scores and sub-scores in the orthostatic intolerance, gastrointestinal, and bladder domains compared with PD patients. Similarly, MSA-P patients had more abnormalities in expiration to inspiration ratio, Valsalva ratio and pressure recovery time than PD patients in objective AFT. With 13.25 as the cut-off score for diagnosis of MSA-P, the total COMPASS-31 score demonstrated high sensitivity (92.6%) and moderate specificity (51.2%) with an area under the curve of 0.765. Based on our results, the COMPASS 31 is an effective tool for evaluation of autonomic function in patients with parkinsonism. The COMPASS-31 could be used as a sensitive and convenient screening tool, especially for the differentiation between MSA-P and PD.

## Introduction

Multiple system atrophy with predominant parkinsonism (MSA-P) and Parkinson’s disease (PD) are different in terms of disease progression and survival. Nevertheless, because MSA-P and PD share similar motor symptoms, differential diagnosis at an early stage is difficult. Although autonomic dysfunction is a characteristic symptom of MSA [1], autonomic function is also commonly involved in PD even from an early stage [2, 3]. Therefore, precise evaluation of autonomic dysfunction is important for diagnosis in patients with parkinsonism. Previous studies have used an objective autonomic function test (AFT) for differentiation of MSA-P in patients with parkinsonism, but optimal evaluation of autonomic dysfunction in patients with parkinsonism is still controversial [4–8]. Additionally, questionnaires on autonomic dysfunction could easily be assessed in the clinic, but only a few studies have investigated the clinical implications of these scales in patients with parkinsonism [7, 8].

The composite autonomic symptom scale 31 (COMPASS 31) questionnaire is a self-rating questionnaire evaluating six domains of autonomic function: orthostatic intolerance, vasomotor, secretomotor, gastrointestinal, bladder, and pupillomotor domains [9]. The COMPASS 31 has already been reported as a useful tool for the evaluation of autonomic dysfunction in various neurologic diseases such as multiple sclerosis, polyneuropathy, and fibromyalgia [10–12]. However, only one previous study has used the COMPASS 31 in MSA and PD patients [7]. This study did not divide MSA patients into predominant subtypes or use the COMPASS 31 for the differential diagnosis of MSA.

In addition, previous studies enrolled PD and MSA-P patients irrespective of dopaminergic medications, thus failing to eliminate the possible confounding effects of such [4–8]. Because dopaminergic medications have various effects on both parkinsonism and autonomic symptoms in patients with parkinsonism [13], the confounding effects due to medications should be minimized when comparing clinical features between patients with MSA-P and PD.

In this study, we investigated the diagnostic significance of the COMPASS 31 score in patients with parkinsonism by comparing the total score and domain scores between drug-naïve patients with MSA-P and PD, and determining the correlation between COMPASS 31 score and AFT results.

## Methods

### Subjects

This study was approved by the Institutional Review Board of Samsung Medical Center, Seoul, Korea and all enrolled subjects provided written informed consent. We recruited drug-naïve patients with PD and probable MSA-P from January 2015 to October 2015 from the movement disorders clinic at Samsung Medical Center. PD was diagnosed based on the UK Brain Bank Criteria for PD [14], and ^18^F-FP-CIT positron emission tomography (PET). Probable MSA-P was determined based on the Second Consensus diagnostic criteria for MSA [1]. Subjects with structural brain lesions, other known neurodegenerative diseases, cognitive impairment (mini-mental status examination score of < 26 or fulfillment of *DSM-IV* criteria for dementia), psychiatric disorders requiring medication, malignancy, or musculo-skeletal problems mimicking parkinsonism were excluded. In addition, we also excluded subjects with medical conditions which could affect AFT results including diabetes mellitus or cardiac arrhythmia. Demographic and clinical data were collected for all patients. Motor symptoms were evaluated with the Unified Parkinson’s Disease Rating Scale (UPDRS) part 3 and by Hoehn and Yahr stage [15].

### Autonomic symptom questionnaire

All enrolled subjects received the COMPASS 31, which is a questionnaire of autonomic symptoms and consists of six domains: orthostatic intolerance, vasomotor, secretomotor, gastrointestinal, bladder and pupillomotor [9]. The six domain scores sum to a total COMPASS 31 score of 0 to 100, and a higher COMPASS 31 score indicates more severe autonomic symptoms.

### Autonomic function test

All enrolled subjects discontinued medications that could affect the AFT results at least 24 hours before the AFT, and refrained from drinking coffee or tea and smoking on the day of the test. Both MSA-P and PD patients underwent our standardized autonomic test battery which included heart rate response to deep breathing (HRDB), the Valsalva maneuver, blood pressure and heart rate response to head-up tilt test (HUT), and sympathetic skin response (SSR). Continuous BP monitoring using the Finometer® (FMS, Amsterdam, the Netherlands), and ECG and respiratory movements were obtained during the HRDB test, Valsalva maneuver, and HUT.

The HRDB test was performed with the patient in a supine position. Patients were asked to breathe deeply by repeatedly inhaling for 5 seconds and exhaling for 5 seconds for 1 minute at a rate of 6 breaths per minute. The difference in heart rate between the end of expiration, end of inspiration and the expiration to inspiration ratio (E:I ratio) were calculated to evaluate cardiovagal function.

The Valsalva maneuver was performed with the patient in a supine position. The patient breathed into a mouthpiece connected to a manometer with an expiratory pressure of 40 mmHg for 15 seconds. Pressure recovery time (PRT) and Valsalva ratio were measured for the assessment of adrenergic and cardiovagal functions, respectively; PRT was defined as the time interval from the bottom of phase III to the complete return of systolic blood pressure to baseline during the Valsalva maneuver [16]. The Valsalva ratio was calculated by dividing the maximum heart rate by the lowest heart rate during the maneuver [17].

HUT was performed on patients after 30 minutes of resting in a supine position on an electrically driven tilt table. Patients were tilted up to 70 degrees for 10 minutes while their blood pressure, heart rate, and the presence of any discomfort, chest pain, dyspnea, dizziness, or syncope was monitored. The presence of orthostatic hypotension (OH) was evaluated as an adrenergic function parameter, which was defined as a fall of at least 30 mmHg in systolic blood pressure and/or a 15 mmHg fall in diastolic blood pressure within two to five minutes of HUT [17].

Sudomotor function was assessed with SSR. SSR was recorded from the palm of the hand and sole of the foot using a Nicolet Viking IV electromyography machine (Nicolet Biomedical, Madison, WI, USA) according to a previously reported protocol [18]. The SSR was considered abnormal if at least one of the two limb responses was absent in five serial stimulation trials [19].

### Statistical analyses

All data were presented as medians and interquartile ranges (IQR). Demographic and clinical data were compared between groups using the Student t-test or Mann-Whitney U-test for continuous variables, and Pearson’s χ^2^ or Fisher’s exact test for categorical variables. To determine the relationship between COMPASS 31 score and AFT results, we performed Spearman’s correlation analysis. Receiver operating characteristic (ROC) curve analysis was performed to establish the optimal cut-off value for the differentiation of MSA-P from PD. A *p*-value < 0.05 was considered significant. Statistical analyses were performed with a commercially available software package (PASW version 18.0; SPSS Inc., Chicago, IL, USA).

## Results

### Patient groups

We recruited 41 patients with idiopathic PD and 27 patients with probable MSA-P. Demographic and clinical details of the enrolled subjects are presented in Table 1. There was no difference between the two groups in demographic data and parkinsonism. Even though MSA-P patients showed higher Hoehn and Yahr stage comparing with PD patients, there was no statistical significance.

**Table 1:** Demographic and clinical characteristics of enrolled subjects with PD and MSA-P.

	PD (n = 41)	MSA-P (n = 27)	*p*-value
**Male/female, n (%)**	20/21 (49/51)	14/13 (52/48)	0.806
**Age, years**	67.0 (57.0–72.0)	69.0 (66.0–74.0)	0.198
**Duration of disease, years**	2.2 (1.0–3.5)	2.0 (1.0–3.0)	0.840
**UPDRS part 3 score**	28.0 (22.0–32.0)	26.0 (19.5–33.0)	0.459
**HY stage 1/2/3, n (%)**	6/32/3 (14.6/78.0/7.3)	3/16/8 (11.1/59.3/29.6)	0.061

PD, Parkinson’s disease; MSA-P, multiple system atrophy with predominant parkinsonism; UPDRS, unified Parkinson’s disease rating scale; HY, Hohen and Yahr.

### Correlation between COMPASS 31 and objective AFT scores

To investigate the efficacy of using the COMPASS 31 for evaluation of autonomic dysfunction in patients with parkinsonism, we performed correlation analyses between COMPASS 31 score and objective AFT results. The total COMPASS 31 score was well-correlated with all objective AFT results, including the E:I ratio, Valsalva ratio, PRT, SSR and OH (S1 Table and S1 Fig). Among the sub-scores of the COMPASS 31, the gastrointestinal domain sub-score was also significantly correlated with all parameters of the objective AFT. Among the objective AFT results, SSR were significantly correlated with the sub-scores of all domains of the COMPASS 31, except for the secretomotor and pupillomotor domains.

### Comparison of the COMPASS 31 and objective AFT between MSA-P and PD patients

**Fig 1:**
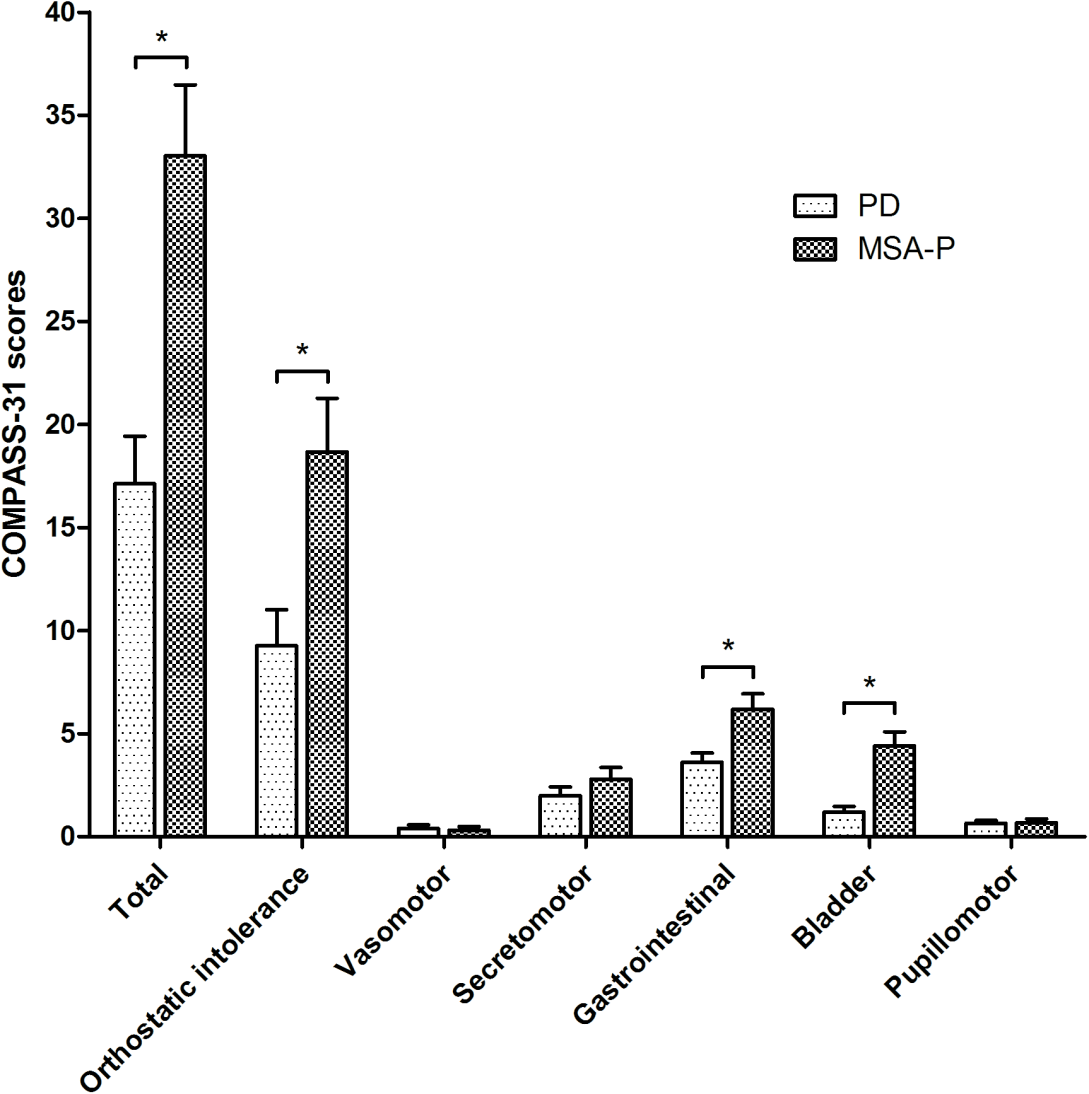
Comparison of COMPASS 31 scores among the patients with PD and MSA-P. The MSA-P patient group had higher total COMPASS 31 scores and higher sub-scores in the orthostatic intolerance, gastrointestinal, and bladder domains compared with PD patients. The error bars represent the standard error of mean. COMPASS 31, composite autonomic symptom scale 31 questionnaire; PD, Parkinson’s disease; MSA-P, multiple system atrophy with predominant parkinsonism. *p-value < 0.05.

MSA-P patients demonstrated more autonomic involvement than PD patients in both COMPASS 31 score and objective AFT results (Table 2). In terms of the COMPASS 31, higher total score and sub-scores for orthostatic intolerance, gastrointestinal, and bladder domains were seen in MSA-P patients compared with PD patients (Fig 1). Similarly, MSA-P patients showed more diffuse and severe autonomic dysfunction than PD patients for E:I ratio, Valsalva ratio, PRT, OH, and SSR among objective AFT parameters.

**Table 2:** Results of the evaluation of autonomic function between PD and MSA-P patients using the COMPASS 31 and objective autonomic function test.

	PD (n = 41)	MSA-P (n = 27)	*p*-value
**COMPASS 31**			
** total score**	12.8 (4.8–27.0)	32.1 (17.5–43.3)	<0.001^a^
** orthostatic intolerance**	8.0 (0–16.0)	20.0 (4.0–32.0)	0.003^a^
** vasomotor**	0 (0–0)	0 (0–0)	0.678
** secretomotor**	0 (0–3.2)	2.1 (0.0–4.3)	0.175
** gastrointestinal**	3.6 (0.4–6.3)	6.3 (3.6–8.9)	0.004^a^
** bladder**	1.1 (0–1.7)	3.3 (1.1–6.7)	<0.001^a^
** pupillomotor**	0.3 (0–1.0)	0 (0–1.3)	0.839
**Objective autonomic function test**			
** E:I ratio**	1.1 (1.0–1.2)	1.1 (1.1–1.1)	0.020^a^
** Valsalva ratio**	1.4 (1.2–1.6)	1.3 (1.2–1.4)	0.032
** pressure recovery time, s**	1.0 (1.0–3.0)	10.0 (3.0–24.0)	<0.001^a^
** orthostatic hypotension, n (%)**	0 (0)	15 (77.8)	<0.001^a^
** abnormality in SSR, n (%)**	5 (12.2)	7 (29.6)	0.106

PD, Parkinson’s disease; MSA-P, multiple system atrophy with predominant parkinsonism; COMPASS 31, composite autonomic symptom scale 31 questionnaire; E:I ratio, expiration:inspiration ratio; OH, orthostatic hypotension; SSR, sympathetic skin reflex.

^a^*p*-value < 0.05

### Sensitivity and specificity of COMPASS 31 for the diagnosis of MSA-P

**Fig 2:**
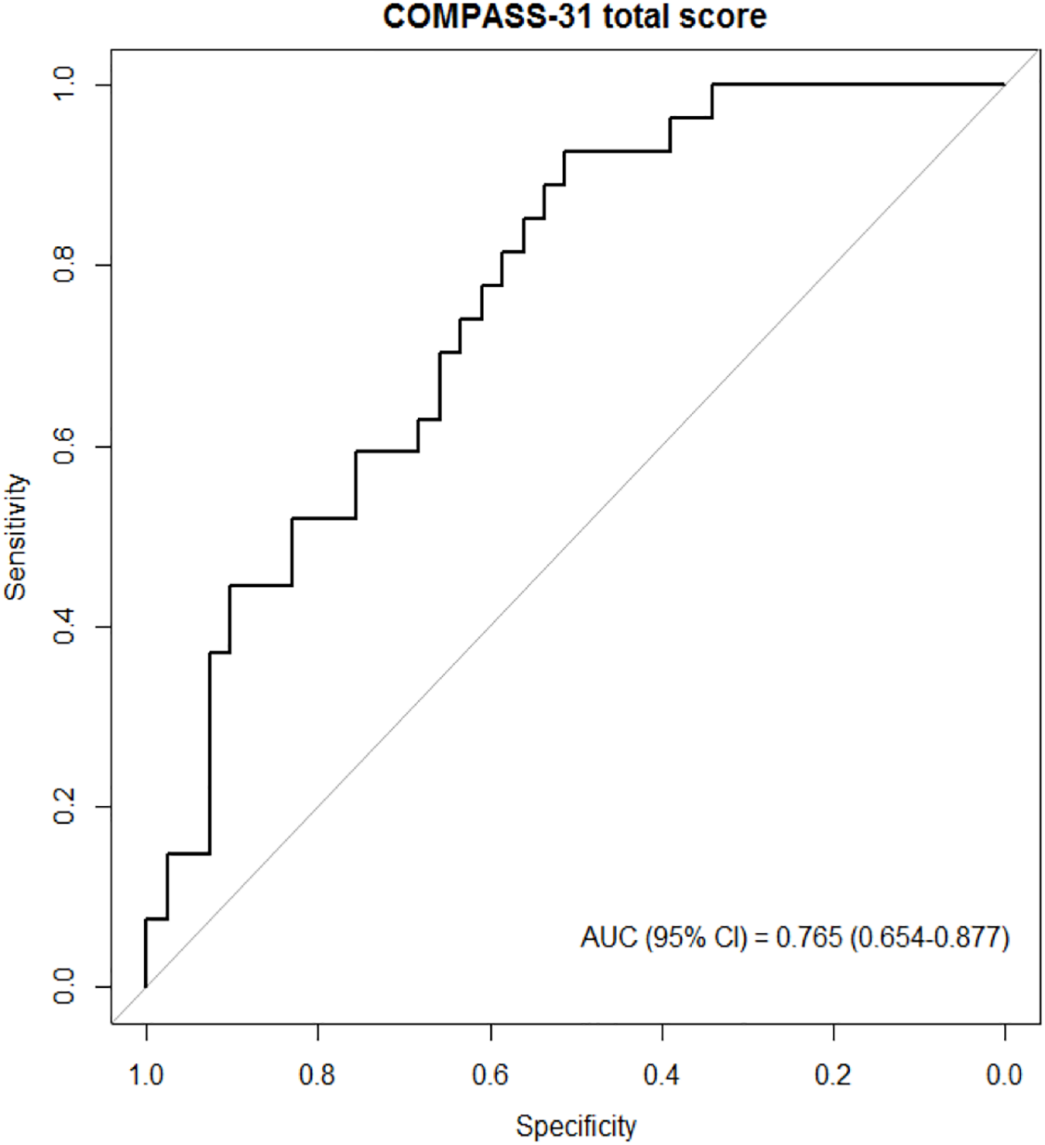
ROC curve for COMPASS 31 total score diagnostic performance. With 13.25 as the cut-off score for differential diagnosis of MSA-P from PD, the total COMPASS 31 score demonstrated high sensitivity (92.6%) and moderate specificity (51.2%) with an area under the curve of 0.765. ROC, receiver operating characteristic; COMPASS 31, composite autonomic symptom scale 31 questionnaire; MSA-P, multiple system atrophy with predominant parkinsonism; PD, Parkinson’s disease.

The sensitivity and specificity of COMPASS 31 total score for discriminating MSA-P from PD (Fig 2 and S2 Table) were 92.6% (75.7–99.1 for 95% CI) and 51.2% (35.1–67.1 for 95% CI), respectively. The area under the curve (AUC) was 0.765 (0.654–0.877 for 95% CI) with a cut-off score of 13.25. In addition, the sub-score for the bladder domain showed a high specificity of 90.2% (76.9–97.3 for 95% CI), moderate sensitivity of 66.7% (46.0–83.5 for 95% CI), and an AUC of 0.789 (0.669–0.909 for 95% CI) with a cut-off score of 3.

## Discussion

This is the first study to investigate the COMPASS 31 for differential diagnosis between MSA-P and PD. Because parkinsonism and autonomic dysfunction are common to both MSA-P and PD, it is difficult to differentiate one from the other. Therefore, precise evaluation of autonomic dysfunction is important and helpful. In this study, COMPASS 31 scores were well-correlated with objective AFT results in patients with parkinsonism. Furthermore, there was a significant difference in COMPASS 31 score between MSA-P and PD patients, and COMPASS 31 total score showed high sensitivity for the diagnosis of MSA-P. Based on these results, the COMPASS 31 could be a convenient and useful tool for screening MSA-P in patients with parkinsonism even from an early stage.

Classically, the objective AFT was regarded as the main tool for diagnosing autonomic dysfunction, but an expensive immovable device and a trained operator are required for this evaluation. Moreover, there is still controversy on the efficacy of using the objective AFT to differentiate between MSA and PD [4–8]. However, autonomic function can be easily assessed in practice with a self-rated questionnaire. The COMPASS-31 has already been reported in previous studies as a useful diagnostic tool for various neurologic diseases [10–12]. In this study, we demonstrated that COMPASS 31 scores were well-correlated with objective AFT results in patients with parkinsonism. Especially, total COMPASS 31 score and the sub-scores for orthostatic intolerance, and gastrointestinal function were significantly correlated with E:I ratio, Valsalva ratio, PRT, OH, and SSR, which had significantly more involvement in MSA-P patients compared with PD patients, except SSR. Therefore, we suggest that the COMPASS 31 could be a useful tool to evaluate autonomic dysfunction in patients with parkinsonism.

When we compared autonomic dysfunction between MSA-P and PD patients using the COMPASS 31, MSA-P patients showed a higher total COMPASS 31 score than patients with PD. Similarly, previous studies also revealed more severe autonomic involvements in MSA-P compared to PD patients [5, 7, 8]. Particularly, MSA-P patients demonstrated higher scores for orthostatic intolerance, gastrointestinal and bladder function than PD patients. In terms of the objective AFT, more severe abnormalities in E:I ratio, Valsalva ratio, PRT, and OH were seen in MSA-P compared with PD patients. In particular, PRT was much longer in MSA-P patients compared with PD patients, and more than 3/4 of subjects with MSA-P showed OH in response to HUT, while no PD patients showed OH. Based on these results, both the COMPASS 31 and objective AFT showed noticeable cardiovascular involvement in MSA-P compared with PD patients. Although a previous study reported cardiovascular autonomic dysfunction in the early stages of PD [2], MSA-P patients have been shown to have more cardiovascular dysfunction than PD patients, which is similar to our results [4]. In addition, subjects with MSA-P showed more gastrointestinal and bladder symptoms based on COMPASS 31 scores compared to patients with PD. Considering that the objective AFT is not a proper tool for evaluating gastrointestinal and bladder function [20], the COMPASS 31 could cover an even wider-spectrum of autonomic dysfunction, and thus could be more helpful for precise evaluation of dysautonomia in patients with parkinsonism.

When we examined the sensitivity and specificity of the COMPASS 31 for the differentiation of MSA-P from PD, COMPASS 31 total score showed high sensitivity but moderate specificity. A previous study using a scoring system based on the objective AFT reported a sensitivity of 89% and specificity of 70% [5], and another study using a battery of cardiovascular autonomic tests showed a sensitivity of 91% and specificity of 94% [4]. Therefore, compared with previous studies [4, 5], we suggest that the total COMPASS 31 score can be used as screening tool for MSA-P in patients with parkinsonism based on the high sensitivity shown in our results.

Despite high sensitivity, the COMPASS 31 demonstrated moderate specificity. Relatively low specificity could result from the recruitment of early, drug-naïve patients in this study, ir from the limitation of COMPASS 31. Considering it is difficult to distinguish PD from MSA-P, especially in early stage, we aimed to investigate the clinical implications of COMPASS 31 for differentiation in patients with early stage. Additionally, to overcome the possible limitation of COMPASS 31, we need instruments/biomarkers with high specificity. In previous studies, the objective AFT showed relatively high specificity compared with our results [4, 5], and the objective AFT could compensate the limitation of COMPASS 31. Moreover, the bladder domain sub-score had high specificity (90.2%) in our study (S2 Table), thus the sub-score of this domain could be also used to overcome the low specificity of COMPASS 31, when using total COMPASS 31 score.

MSA is usually divided into two subtypes based on the predominant symptoms: parkinsonism (MSA-P) and cerebellar ataxia (MSA-C). Unlike previous studies [6, 7], we only enrolled patients with PD and MSA-P and did not enroll patients with MSA-C because it is difficult to differentiate MSA-P, but not MSA-C, from PD in the early stages. Additionally, when comparing autonomic dysfunction between MSA and PD patients, it is important to minimize possible confounding factors. Considering that the autonomic nervous system is usually more involved in disease progression, we enrolled MSA-P and PD patients with similar disease severity. However, because the main symptom of MSA-C is ataxia, not parkinsonism, it is difficult to match the disease severity of MSA-C and PD. Furthermore, we recruited drug-naïve MSA-P and PD patients in our study to minimize other possible confounding factors due to dopaminergic medications.

This study has some limitations. First, our sample size was relatively small. However, even with a small sample size, we found that the COMPASS 31 was a useful tool for evaluating autonomic function in patients with parkinsonism and could be used as a screening tool for MSA-P. Additionally, we enrolled drug-naïve, early stage subjects with MSA-P and PD. Considering clinical diagnosis could be changed with follow up, there is a possibility for changed diagnosis. However, all the PD patients in our study showed excellent response to dopaminergic medication and were also confirmed with dopamine transporter PET. Another limitation is that our study was cross-sectional. Since autonomic involvement could be more prominent with disease progression, it is important to investigate further with longitudinal follow-up studies. In this study, we focused on the utility of the COMPASS 31 in patients with parkinsonism since the differential diagnosis between MSA-P and PD is usually difficult in the early stages of these diseases. Moreover, we recruited drug-naïve MSA-P and PD patients so we could eliminate possible confounds due to medications or disease severity. Lastly, we did not enroll normal controls. In this study, our primary objective was to determine the efficacy of the COMPASS 31 in patients with parkinsonism, thus we enrolled patients with MSA-P and PD, two of the most common and dominant diseases in parkinsonism.

In conclusion, the COMPASS 31 was a convenient and effective tool for the evaluation of autonomic function in patients with parkinsonism along with the objective AFT. Furthermore, considering that the total COMPASS 31 score showed high sensitivity for differentiating between MSA-P and PD, this questionnaire could be a helpful tool for screening MSA-P from parkinsonism.

## Supporting information

S1 Fig Correlations between the total COMPASS 31 score and objective autonomic function test results in all enrolled subjects. Spearman correlation analysis was performed to determine the relationship between the total COMPASS 31 score (y-axis) and E:I ratio, Valsalva ratio, and pressure recovery time (x-axis). The total COMPASS 31 score showed negative correlations with the E:I ratio (A) and Valsalva ratio (B). A significant positive correlation was observed between the total COMPASS 31 score and pressure recovery time (C).
(TIF)

S1 Table Correlation analysis between COMPASS 31 scores and objective autonomic function test results. E:I ratio, expiration:inspiration ratio; PRT, pressure recovery time; OH, orthostatic hypotension; SSR, sympathetic skin reflex. *p*-values below 0.029 are considered significant and marked with an asterisk based on Bonferroni corrections.
(DOCX)

S2 Table Discriminant analysis results using the COMPASS 31 score for differential diagnosis of MSA-P from PD. COMPASS 31, composite autonomic symptom scale 31 questionnaire; MSA-P, multiple system atrophy with predominant parkinsonism; PD, Parkinson’s disease; CI, confidence interval; AUC, area under the curve.
(DOCX)
