# Supplementary figures and images for: The composite autonomic symptom scale 31 is a useful screening tool for patients with Parkinsonism
Source: PLoS One. 2017 Jul 6;12(7):e0180744. doi: 10.1371/journal.pone.0180744 (PMC5500372; doi:10.1371/journal.pone.0180744)

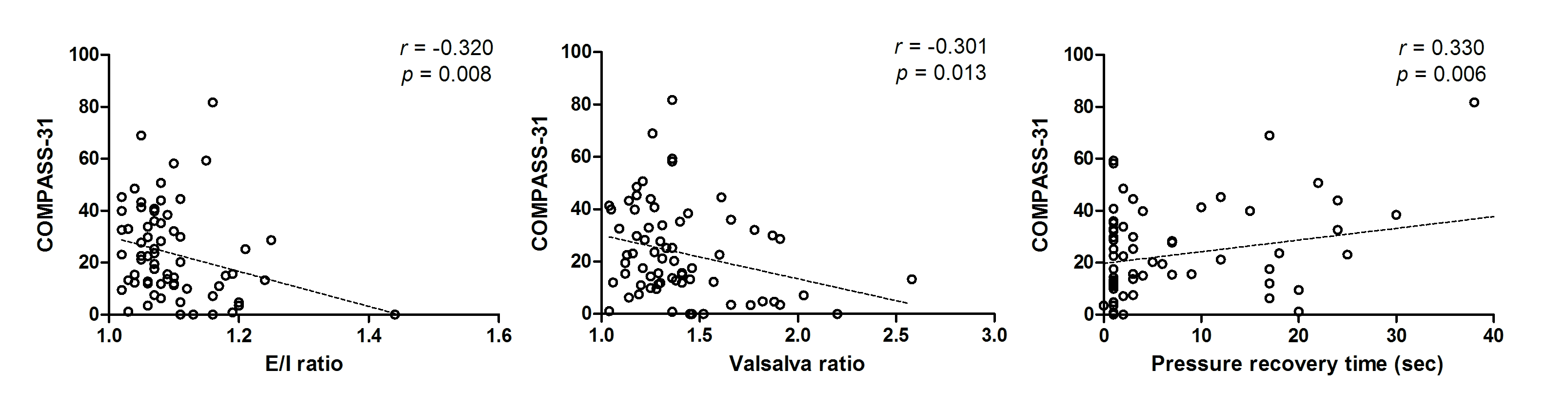

Supplement: S1 Fig — Spearman correlation analysis was performed to determine the relationship between the total COMPASS 31 score (y-axis) and E:I ratio, Valsalva ratio, and pressure recovery time (x-axis). The total COMPASS 31 score showed negative correlations with the E:I ratio (A) and Valsalva ratio (B). A significant positive correlation was observed between the total COMPASS 31 score and pressure recovery time (C). (TIF) [file pone.0180744.s001.tif]
