# Supplementary material for: The composite autonomic symptom scale 31 is a useful screening tool for patients with Parkinsonism
Source: PLoS One. 2017 Jul 6;12(7):e0180744. doi: 10.1371/journal.pone.0180744 (PMC5500372; doi:10.1371/journal.pone.0180744)
Supplement: S1 Table — E:I ratio, expiration:inspiration ratio; PRT, pressure recovery time; OH, orthostatic hypotension; SSR, sympathetic skin reflex. p-values below 0.029 are considered significant and marked with an asterisk based on Bonferroni corrections. (DOCX) [file pone.0180744.s002.docx]

|  | Objective autonomic function test | | | | |
| --- | --- | --- | --- | --- | --- |
|  | E:I ratio | Valsalva ratio | PRT | OH | SSR |
| COMPASS 31 |  |  |  |  |  |
| total score | -0.320* | -0.301* | 0.330* | 0.408* | 0.402* |
| orthostatic intolerance | -0.204 | -0.234 | 0.239* | 0.325* | 0.332* |
| vasomotor | -0.230 | -0.204 | 0.030 | -0.065 | 0.260* |
| secretomotor | -0.071 | -0.005 | 0.023 | 0.222 | 0.154 |
| gastrointestinal | -0.451* | -0.354* | 0.420* | 0.328* | 0.277* |
| bladder | -0.180 | -0.156 | 0.325* | 0.432* | 0.396* |
| pupillomotor | -0.066 | -0.038 | -0.075 | 0.095 | 0.150 |
