# Supplementary material for: The composite autonomic symptom scale 31 is a useful screening tool for patients with Parkinsonism
Source: PLoS One. 2017 Jul 6;12(7):e0180744. doi: 10.1371/journal.pone.0180744 (PMC5500372; doi:10.1371/journal.pone.0180744)
Supplement: S2 Table — COMPASS 31, composite autonomic symptom scale 31 questionnaire; MSA-P, multiple system atrophy with predominant parkinsonism; PD, Parkinson’s disease; CI, confidence interval; AUC, area under the curve. (DOCX) [file pone.0180744.s003.docx]

|  | Sensitivity  (95% CI) | Specificity  (95% CI) | AUC  (95% CI) | cut-off  value |
| --- | --- | --- | --- | --- |
| Total score | 92.6% (75.7-99.1) | 54% (0.37-0.69) | 0.765 (0.654-0.877) | 13.25 |
| Orthostatic intolerance | 66.7% (46.0-83.5) | 73.2% (57.0-85.8) | 0.706 (0.577-0.836) | 4 |
| Vasomotor | 3.7% (0.0-19.0) | 97.6% (87.1-99.9) | 0.482 (0.401-0.564) | 5 |
| Secretomotor | 66.7% (46.0-83.5) | 51.2% (35.1-67.1) | 0.592 (0.460-0.724) | 1 |
| Gastrointestinal | 63.0% (42.4-80.6) | 73.2% (57.1-85.8) | 0.706 (0.574-0.839) | 7 |
| Bladder | 66.7% (46.0-83.5) | 90.2% (76.9-97.3) | 0.789 (0.669-0.909) | 3 |
| Pupillomotor | 11.1% (2.4-29.2) | 95.1% (83.5-99.4) | 0.486 (0.352-0.620) | 7 |
